# A Quantitative Detection Algorithm for Multi-Test Line Lateral Flow Immunoassay Applied in Smartphones

**DOI:** 10.3390/s23146401

**Published:** 2023-07-14

**Authors:** Shenglan Zhang, Xincheng Jiang, Siqi Lu, Guangtian Yang, Shaojie Wu, Liqiang Chen, Hongcheng Pan

**Affiliations:** 1 Key Laboratory of Advanced Manufacturing and Automation Technology (Guilin University of Technology), Education Department of Guangxi Zhuang Autonomous Region, Guilin 541006, China; zsl@glut.edu.cn (S.Z.); 2120211105@glut.edu.cn (X.J.); wrmcake@163.com (S.W.); m18869047581@163.com (L.C.); 2 School of Information Science and Engineering, Guilin University of Technology, Guilin 541006, China; 2120211037@glut.edu.cn; 3 Guangxi Key Laboratory of Electrochemical and Magneto-Chemical Functional Materials, College of Chemistry and Bioengineering, Guilin University of Technology, Guilin 541004, China; zzzzzruo@163.com

**Keywords:** lateral flow immunoassay, immunosensors, machine vision, support vector machine, smartphone application

## Abstract

The traditional lateral flow immunoassay (LFIA) detection method suffers from issues such as unstable detection results and low quantitative accuracy. In this study, we propose a novel multi-test line lateral flow immunoassay quantitative detection method using smartphone-based SAA immunoassay strips. Following the utilization of image processing techniques to extract and analyze the pigments on the immunoassay strips, quantitative analysis of the detection results was conducted. Experimental setups with controlled lighting conditions in a dark box were designed to capture samples using smartphones with different specifications for analysis. The algorithm’s sensitivity and robustness were validated by introducing noise to the samples, and the detection performance on immunoassay strips using different algorithms was determined. The experimental results demonstrate that the proposed lateral flow immunoassay quantitative detection method based on image processing techniques achieves an accuracy rate of 94.23% on 260 samples, which is comparable to the traditional methods but with higher stability and lower algorithm complexity.

## 1. Introduction

The colloidal gold immunoassay is a highly specific and efficient immunological analysis technique that employs colloidal gold as a marker and integrates with nitrocellulose membrane chromatography [1]. This method yields results within minutes without the need for complex instruments or cumbersome procedures, making it ideal for on-site detection. Moreover, it offers high sensitivity and low cost, and has crucial applications in medical diagnosis [2], food safety [3], and environmental monitoring [4].

The traditional colloidal gold immunoassay [5] relies on visual interpretation by human eyes, based on the coloration of the test line and control line, and can only provide qualitative negative or positive results. Recently, the development of multi-test line and graded stepwise colloidal gold immunoassay methods has enabled semi-quantitative analysis based on the number of colored lines [6]. However, the visual assessment of these methods is often subjective and lacks accuracy, failing to meet the high precision requirements in applications such as tumor diagnosis [7] and drug concentration detection [8]. Subsequently, colorimetric methods [9] have been introduced to achieve quantitative analysis by comparing with known concentrations of standard substances. Yet, these methods also depend on visual interpretation, thus remaining prone to human errors. To eliminate errors from manual judgment, research has turned to reading devices to obtain the intensity or signal change of the lines. By extracting the characteristic information and comparing it with a known concentration standard curve, the concentration of the target substance can be obtained.

In recent years, researchers have increasingly studied the use of computer vision technology for immunoassay detection. However, graded LFIA strips present certain challenges, such as cross-interference [10] and peak overlap [11], which result in decreased detection accuracy and reliability. Therefore, developing efficient methods for processing the images of graded LFIA strips to improve quantitative accuracy and reliability is an important research direction and a promising way forward.

Image processing technology is a powerful tool for optimizing digital images [12]. Techniques such as removal of backgrounds and noise, enhancement of detection sensitivity and specificity, and segmentation of target bands can improve the image quality and quantification of graded LFIA strips. Deep learning algorithms [13], a machine learning method, can automatically recognize different test lines on an LFIA strip and perform classification and quantitative analysis. These technologies provide new ways and methods for analyzing and detecting graded LFIA strips, thereby improving detection accuracy and reliability. However, a substantial amount of data are required to train these algorithms and ensure validity.

Recent research studies have focused on developing novel technologies for the quantification of immunochromatographic assays. For example, Zeng et al. (2021) developed an image segmentation method that uses gold LFIA strips for quantitative analysis [14]. This method reduces image noise caused by environmental factors, solves problems with irregular and fuzzy boundaries between the control line and the test line, and improves recognition errors caused by shallow test lines. Han et al. (2020) proposed a low-cost, highly sensitive lateral flow biosensing platform based on soluble polymer mixtures [15], which is combined with a smartphone-based reader for high-performance point-of-care testing. Rong et al. (2020) integrated fluorescence lateral flow detection, detection boxes, and signal detection devices, resulting in a multi-channel LFIA quantitative reader that can be used for high-throughput screening of infectious diseases [16].

In this study, we developed an intelligent detection algorithm based on image processing techniques, with the following main contributions:

(1) A dark box was designed to reduce the impact of ambient light on image acquisition, and differences in image quality obtained with different models of smartphone were compared.
(2) An intelligent algorithm was developed that utilizes binarization to identify the darker control line and detects the test line through operations such as image denoising, pixel accumulation, and convolution.
(3) Machine learning methods were employed to fit the relationship between feature values and concentration, thereby validating the accuracy of the algorithm. The algorithm’s effectiveness under various conditions was verified by adding noise to the images. Furthermore, a comparison of the current algorithm versus other detection algorithms, ranging from traditional algorithms to machine learning and deep learning approaches, was conducted.

## 2. Materials and Methods

### 2.1. Experimental Materials and Instruments

**Figure 1:**
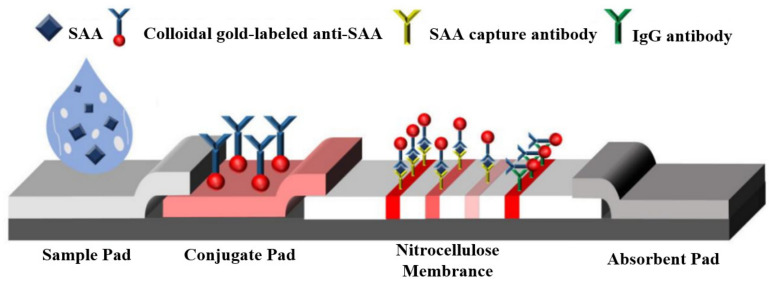
Test strip schematic diagram.

In normal human conditions, the concentration of serum amyloid A protein (SAA) is typically below 10 μg/mL. However, during inflammatory infections, SAA concentration can increase by 1000 times within the acute phase response (APR) period of 24 to 72 h. Given this characteristic, SAA was selected as the research subject for this study’s experiments. Lateral flow immunoassay quantitative detection requires materials and instruments that typically include the following: immunoassay test strips consist of a paper base, a probe, labeling agents, and other components. As shown in Figure 1, these strips utilize chemical reactions to convert target substances into visible color signals. Sample preparation involved using bovine serum albumin (BSA), which was purchased from Hefei Bomei Biotechnology Co. (Hefei, China) The SAA monoclonal antibody pair and SAA antigen used for labeling and capturing were obtained from Wuhan Huamei Biological Engineering Co. (Wuhan, China). Colloidal gold (25 nm) was synthesized in the laboratory, and glass cellulose membranes and polyvinyl chloride backing plates (PVC plates) were purchased from Shanghai Jining Biotechnology Co. (Shanghai, China).

**Figure 2:**
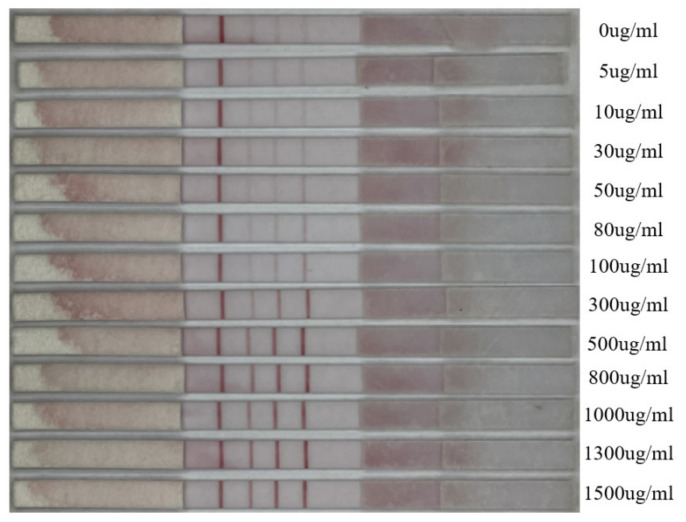
Example of samples at different concentrations.

In this study, we conducted experiments using SAA test strips. We prepared samples with different concentrations by diluting the standard solution 700 times. The concentrations included 0, 5, 10, 30, 50, 80, 100, 300, 500, 800, 1000, 1300, and 1500 μg/mL, totaling 260 samples. Each concentration had 20 samples. We chose such a wide concentration gradient to have more data points for better curve fitting and calibration when the samples’ color changed drastically. An example of samples at different concentrations is shown in Figure 2. The experiments were conducted at a temperature of 25 °C and a humidity of 50%. After adding the samples, we waited for 5 min before collecting the samples for feature extraction.

**Figure 3:**
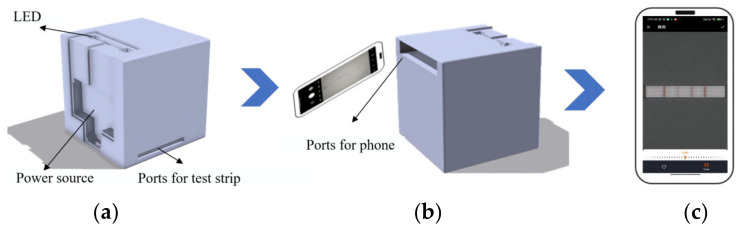
(**a**) Design drawing of the front of the dark box. (**b**) Design of the mobile phone slot. (**c**) Example diagram for collecting samples.

Thakur et al. (2021) investigated the impact of flash usage while capturing images with smartphones [17]. They found that enabling the flashlight resulted in a higher recognition accuracy as the built-in flashlight of smartphones reduced the impact of ambient light. However, different models of smartphone flashlights also exhibit variations, which can introduce errors that are difficult to correct. To control the lighting conditions impacting test strip detection, we utilized a dark box to provide a stable lighting environment, thus ensuring the accuracy and reproducibility of image acquisition. The dark box shell was made of plastic material, sealed internally, and colored using black dye. It features a black frosted glass surface as the platform for placing test strips, which is characterized by a low reflection of light. A white LED light (wavelength of 450 nm, color temperature of 3000 K, power of 4.8 watts/meter, luminous flux of 450 LM, and power supply of 12 V) was installed inside the dark box to replace the smartphone flashlight. This not only reduces the influence of natural light but also addresses the issue of different smartphone flashlight models. Both the test strips and the smartphone being used were inserted into the dark box, and the structure and usage of the dark box are demonstrated in Figure 3.

Three common smartphone models were selected for the experiments: Xiaomi 11, iPhone 12, and Huawei P30. The parameters of these three models are shown in Table 1. All three smartphones have a high camera performance and high popularity in China. Under consistent lighting, images were acquired using each smartphone to evaluate color value differences and to assess the practicality and applicability of the proposed detection method.

### 2.2. Experimental Procedure

**Figure 4:**
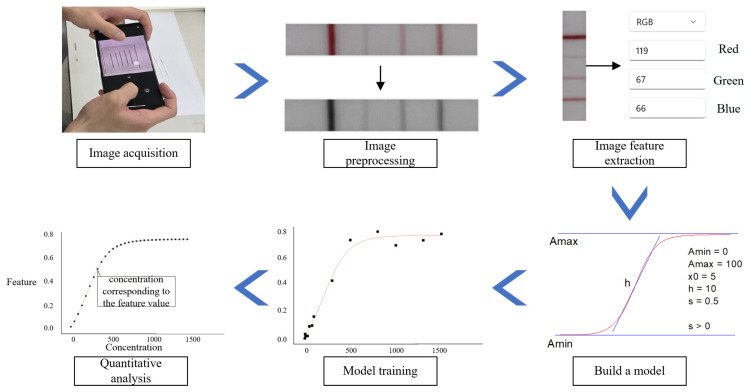
The overall process of lateral flow immunoassay quantitative detection.

To ensure high-quality and repeatable data, multiple factors were considered when preparing the samples. When using a smartphone to collect samples, a 20 cm shooting distance and a horizontal angle were used. The optimal shooting environment should have a humidity lower than 50% and a temperature range of 20 °C to 28 °C. Following sample collection, we utilized the OpenCV function library to preprocess the raw images for improving the accuracy of image feature values, applying steps such as noise reduction and contrast enhancement. Subsequently, smart algorithms were employed for feature value extraction, followed by mathematical fitting of the feature values with the test strip’s concentration. Support vector machine (SVM) [18] is an effective machine learning method that showed the best performance in the fitting process. With the model training complete, the proposed algorithm could then be used for recognition and quantitative analysis of new data. The overall process of lateral flow immunoassay quantitative detection is shown in Figure 4.

### 2.3. Algorithm Design

**Figure 5:**
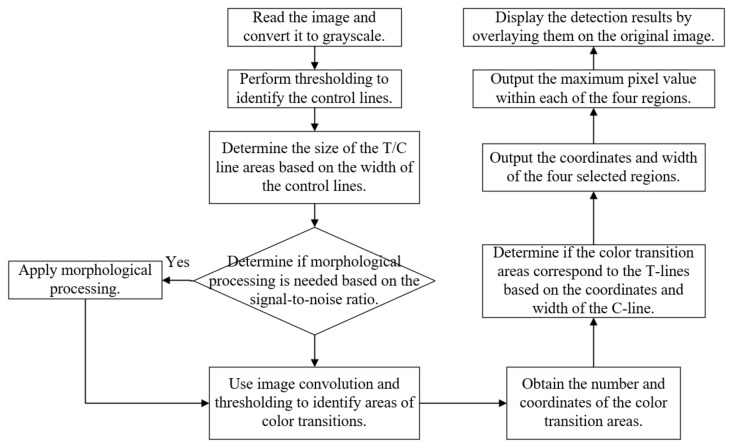
Overall flowchart of the algorithm.

The overall flow of the algorithm is shown in Figure 5:

- Step 1: Using threshold segmentation to find the control line

**Figure 6:**
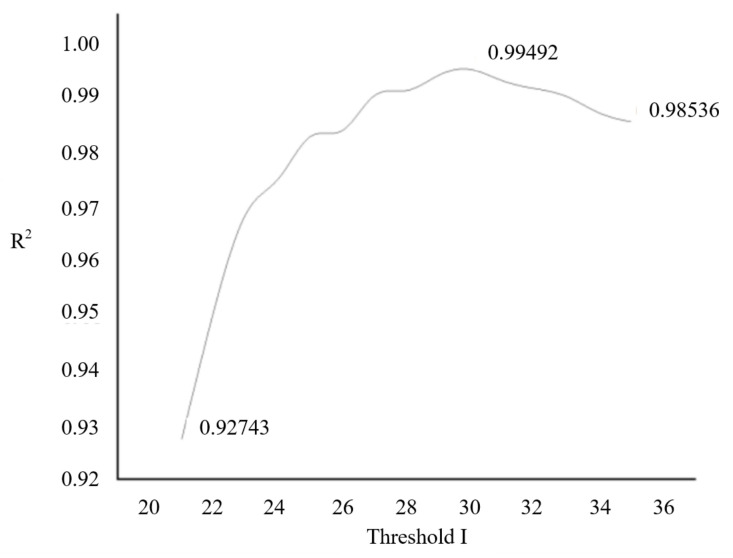
Graph showing the reasons for choosing threshold value I.

Control lines on immunochromatographic strips tend to be dark and stable, and in this case, a binarization method is used to find the control line with good results. To determine the sum of pixel values for each column in the image, the following formula is used:

(1) $$S_{j}=\sum_{i=1}^{h}I_{i,j}$$

where *S_j_* is the sum of pixel values in the *j*th column, and *I_i,j_* is the pixel value at position [*i,j*]. To obtain a threshold for binarization, the minimum pixel value in this one-dimensional image is added to an external value *I* = 30 (as determined from Figure 6 below). This result is then multiplied by the image height to yield the threshold:

(2) $$Threshold={\left(MinValue+I\right)}^{H}$$

where *MinValue* is the minimum pixel value in the one-dimensional image that changes according to the pixel value of the image; and *i* is an externally set value. The *MinValue* and *I* are added together and multiplied by *H*. The result is the binarized threshold, which is just enough to segment the control line at this point.

The left half of the image is horizontally flipped and then multiplied, mirroring the image for comparison. This process determines the width of the control line and obtains the position of the rightmost end of the control line. If the width of the control line is greater than 5 (a threshold set based on experimental experience to ensure algorithm performance and accuracy) and the numerical value of the rightmost end is greater than 0, the control line has successfully been segmented and detected. Subsequently, the width and position results are then output.

- Step 2: Denoise processing

**Figure 7:**
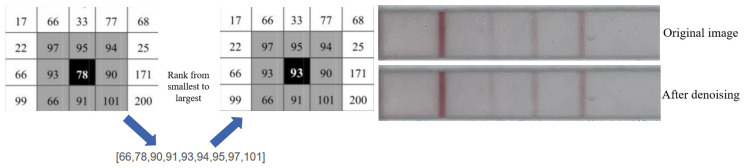
Median filtering principle and an example of the effect.

After zooming in on the image of the test strip taken by the smartphone, we noticed that there are noises between the lines. Unfortunately, these noises are not fully eliminated by threshold segmentation in areas where the test strip is lighter in color. To address this, we implemented a denoising algorithm. After several experiments, median filtering was selected as the optimal method, which could effectively remove noises while retaining the image edge and detail information, thus improving reliability and accuracy. The median filtering results are shown in Figure 7.

**Figure 8:**
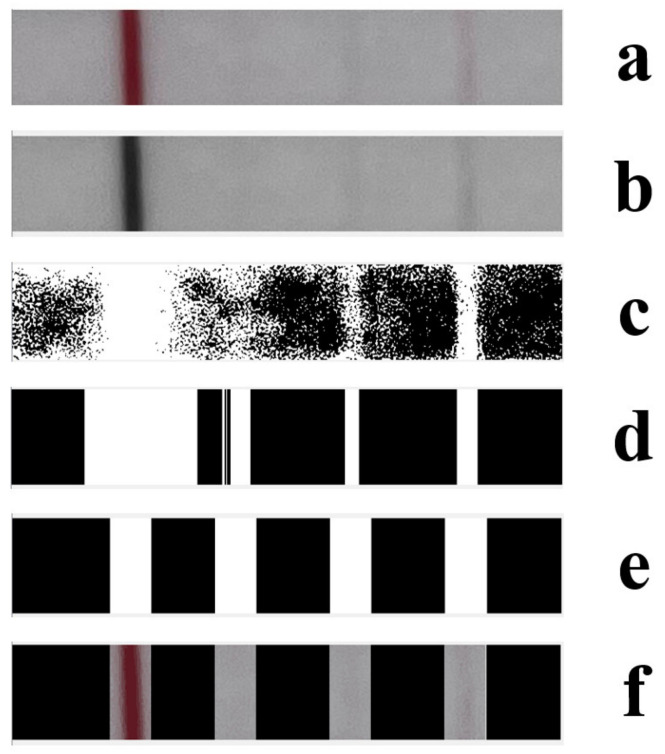
(**a**) Original image; (**b**) grayscale image; (**c**) binarized image after denoising; (**d**) rough area partitioning; (**e**) fine area partitioning; and (**f**) final result image.

To binarize the image, the image pixel average is multiplied by a factor to determine the threshold, which is applied to the left half. This factor is reduced by 0.05, and pixel accumulation on the left half is used to calculate the signal-to-noise ratio (SNR). An SNR below 0.75 indicates significant remaining noise, which is addressed using morphological operations. The binarization results after noise removal are shown in Figure 8c.

- Step 3: Use of convolution and binarization to locate test lines

When locating the control lines, the left half of the image is isolated and flipped horizontally. This flipped section is then subjected to dot-product operation with the initial unflipped region before the column vectors in the photo are summed into an array. A convolution operation is performed between this vector array and the result of the earlier dot-product operation:

(3) D(x,y)=I_left(x,y)×I_flipped(x,y)

(4) $$C(K)=\sum_{}^{}D(x,k)\times I\_column(x)$$

In these calculation processes, *I_left(x,y)* indicates the left half portion of *I(x,y)* image, adopting image values of *I(x,y)* on the image’s left side while setting all other values as 0. *I_flipped(x,y)* refers to a horizontal flip operation of *I(x,y)* image, specifically represented by *I(width-x,y)* wherein width is the image width. C(K) represents the *k*-th element in the convolution results. *D(x,k)* denotes the *k*-th column of symmetry detection image *D(x,y)*. *I_column(x)* describes the *x*-th column of image *I(x,y)*. The Σ sign signifies the summation operations. 

Next, we binarize the image using the pixel mean multiplied by 1.5 (determined through repeated experimentation) as the threshold value for evaluation. Pixel values surpassing the determined threshold are reset to 0, while those below it are set to 1. Finally, we convert the input image into a matrix form and record the position within the end list, where there exist sudden color changes as indicated by the 0 to 1 transition points on the right-side markings of the test lines. These positions are similarly identified within the left markings of such test lines via the head list’s recording. The resulting outputs are illustrated in Figure 8d.

- Step 4: Accurate Selection of Control and Test Lines

Based on the recorded color transition positions of the left and right portions of the control and test lines, we obtain two lists—head and end. Next, we compare the width measurements of the head versus the end lists. If they are similar in width, we traverse the end, identify specific points, and eliminate their respective values within the head and end lists. For the control lines, the values are reduced to their rightmost position, and the width is utilized as a reference point to help determine positioning within both head and end listings. Subsequently, we inspect the end locations, verifying whether the distance between the subsequent head and the current end positions falls two times below the input value. The positions within the end list are then deleted, and the corresponding values within the head list are shifted one place to the right. The purpose of this step is to further verify the authenticity of the test lines, and the final result is shown in Figure 8e.

- Step 5: Further optimizing the location of test and control lines

Initially, if the length of the end list exceeds 4, additional lines require removal and further adjustment. Alternatively, if the length of the head list is 3 and meets specific size conditions, head(1) and end(1) values are removed. Additionally, further calculations are applied to the head and end lists beyond the first grouping. Lengthier lists are broadened by a factor that is three times the control line’s width, sorted in size order, and utilized for new value assignments within the head and end lists. If the length of the head list is 2 or 3, we take the midpoint reading of head(0), end(0), head(1), and end(1) to obtain new values as calculated according to established methodologies. Finally, the listings for the head and the end indicate the precise locations of the test and control lines within the input image, which are later incorporated into the original image, as shown in Figure 8f.

We extract four regions and apply Gaussian blur to them, calculating the maximum value within each area. Gaussian blur is computed using normal distribution mathematics to change every pixel in the image:

(5) $$G(r)=\frac{1}{{\sqrt{2\pi \sigma^{2}}}^{N}}e^{-r^{2}/(2\sigma^{2})}$$

In this process, *r* refers to the blur radius, while *σ* is the standard deviation of the normal distribution. We compare the maximum value to the average value of the remaining parts (*ARG*). If *ARG* is greater than the maximum value, we replace the maximum value with the average value. The four values from left to right represent the control line *C*, test line *T*1, test line *T*2, and test line *T*3, respectively.

The final calculated results for the four lines and the two lines’ read are as follows:

(6) Y1=(T1+T2+T3−3×ARG)/C

(7) Y2=(T1−ARG)/C

### 2.4. Fitting Eigenvalues versus Concentration

As *Y*1 represents the reading value for the four lines, it better reflects the detection performance of the algorithm for multi-line test strips. Therefore, we extracted five sets of samples for the *Y*1 and concentration values, as shown in Table 2:

Support vector machine is a supervised learning model used for classification and regression analysis. Its core concept centers on finding hyperplanes that map sample features to high-dimensional spaces and segmenting them correspondingly. Employing SVM allows us to establish nonlinear relationships between feature values and concentration values.

Using SVM, we constructed an optimization problem to seek out the optimal hyperplane positioning that evenly segregates all data points while maintaining maximal distance from either side of the grouping. Data points within close proximity to the hyperplane are referred to as “support vectors”. Their role is to collectively define the decision boundary when classifying new data. The following equation clarifies this:

(8) $$min_{w,b,\xi }\frac{{\left|\left|w\right|\right|}^{2}}{2}+C\sum_{i}^{}\xi_{i}$$

Here, *w* represents the weight vector and b refers to the bias term. $\zeta_{i}$ indicates the distance between sample *i* and the hyperplane. The purpose of this equation is to minimize model complexity with regard to misclassifications while finding a segmentation hyperplane that classifies all training samples accordingly. The “soft margin” allows some points to be around or even exceeding the margin boundary to better fit the relationship between feature values and outcomes. *C* is a hyperparameter that regulates model complexity and penalizes wrong classifications. By training the appropriate model, we can fit the test sample data into it and predict their respective concentration values. It is noteworthy that SVM performs well when solving issues with small samples and nonlinear data distribution while demonstrating high reliability and stability. Therefore, combining the results from our experiments in this study, we opted to utilize an SVM algorithm for fitting feature values to concentration values to achieve optimal effects.

When using SVM to fit data, the goodness of fit, *R*^2^ value, is a commonly used statistical metric for evaluating the fitting performance of the model. The calculation of *R*^2^ is based on the total sum of squares, the regression sum of squares, and the residual sum of squares, where the regression sum of squares represents the ability of the fitted model to explain the variance in the data, the total sum of squares represents the total variance in the data, and the residual sum of squares represents the variance that the fitted model fails to explain. Therefore, the closer *R*^2^ is to 1, the stronger the explanatory power of the fitted model for the data, and the better the fit. The formula for calculating *R*^2^ is follows:

(9) $$R^{2}=1-\frac{SS_{res}}{SS_{tot}}$$

Here, *SS_res_* stands for the residual sum of squares, and *SS_tot_* represents the total sum of squares. The specific calculation formula is as follows:

(10) $$SS_{tot}=\sum_{i=1}^{n}(y_{i}-\bar{y}{)}^{2}$$

(11) $$SS_{res}=\sum_{i=1}^{n}(y_{i}-f_{i}{)}^{2}$$

*SS_tot_* has two main sources: (1) diverse input values *x_i_* leading to broad output values *y_i_*, and (2) random errors. *f_i_* denotes the value on the regression line.

## 3. Data Analysis and Discussion

### 3.1. Image Differences under Different Phones

**Figure 9:**
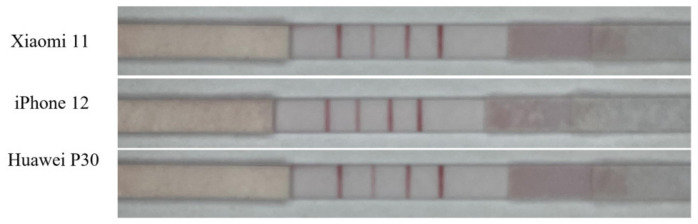
An example of the three phones shooting the same concentration.

**Figure 10:**
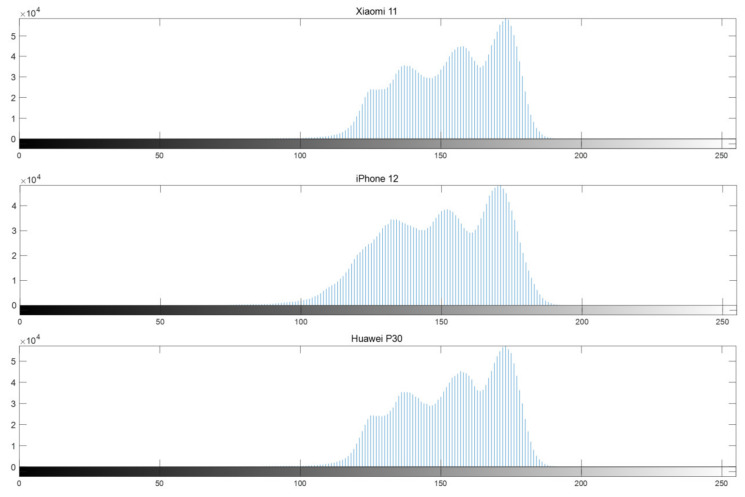
Histogram of pictures taken by three smartphones.

We extracted a sample of 1000 μg/mL from the samples captured using Xiaomi 11, iPhone 12, and Huawei P30 smartphones during the same period. An example diagram is shown in Figure 9. To observe the overall pixel distribution, we calculated the histogram. Histogram of pictures is shown in Figure 10. By analyzing the histogram, we could observe differences in color distribution, contrast, and saturation among the three smartphones.

The histogram analysis showed minimal differences in color distribution, contrast, and saturation between the Xiaomi 11 and Huawei P30 smartphones. However, the images taken by the iPhone 12 smartphone show detection values earlier on the *x*-axis, with a less pronounced first histogram peak. This suggests that the iPhone 12 is capable of capturing low-light details, such as details in darker or shadowed areas, more effectively. Additionally, its image displays a more even overall brightness distribution. These differences may be attributed to various factors, including different image processing algorithms, camera sensors, and software optimization employed by each smartphone.

### 3.2. Results of Fitting

**Figure 11:**
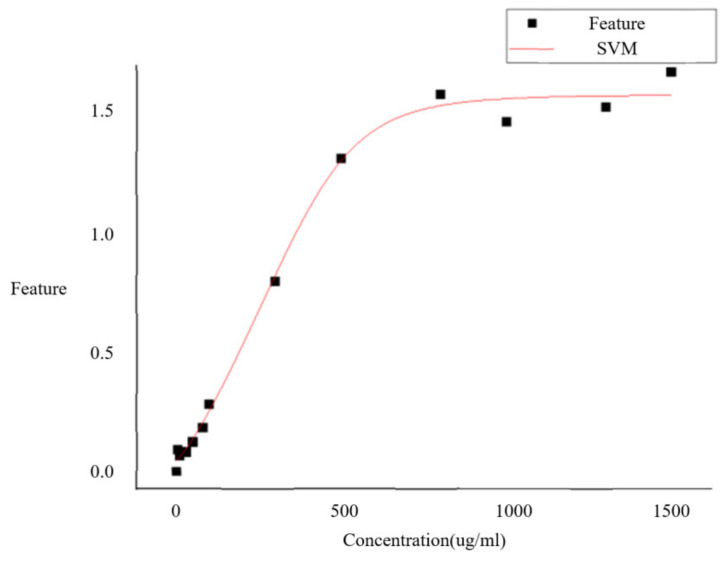
SVM fitting of feature values and concentrations.

The fitted plot is shown in Figure 11. The *R*^2^ value of the fitting equation is 0.99492, indicating that the model has a strong explanatory power over the sample data. Additionally, we calculated the conventional residuals of the model, and the results are shown in the graph. These results suggest that the model has a good predictive performance and high reliability. These findings further validate the fitting model’s adaptiveness and accuracy to the sample data, providing a reliable foundation for subsequent data analysis.

**Figure 12:**
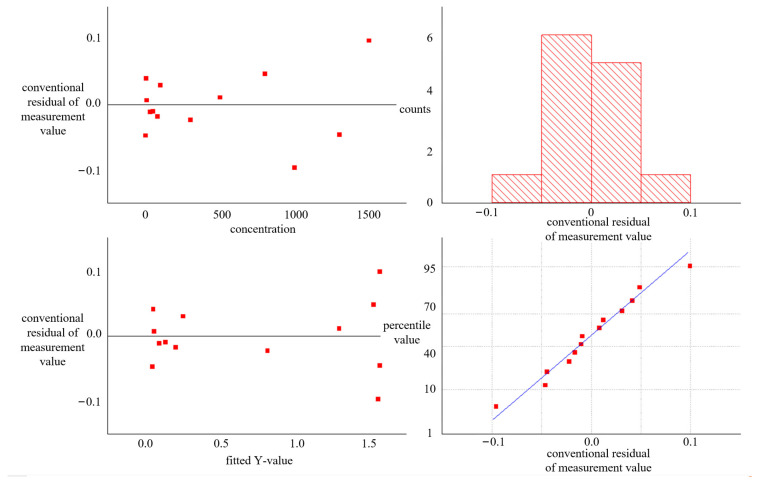
Fitting error parameter chart.

This study utilized SVM to fit the sample data and calculated the *R*^2^ value as an evaluation index. The results indicate that the model has a strong explanatory power over the data, with a good fitting effect and an *R*^2^ value of 0.99492. Furthermore, by calculating the total sum of squares, the regression sum of squares, and the residual sum of squares, we found that the conventional residuals’ interval is within [−0.1, 0.1]. Fitting error parameter chart is shown in Figure 12. Subsequent analysis confirmed that computer vision technology holds wide application prospects in lateral flow immunoassay detection and can significantly improve detection accuracy and reliability. However, further exploration is necessary to enhance algorithm performance and stability to meet practical application needs.

### 3.3. Algorithm Performance Testing

**Figure 13:**
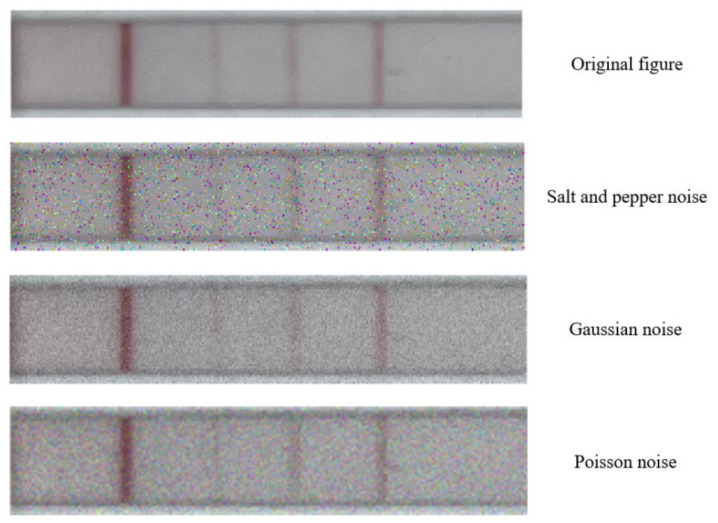
Example of adding salt-and-pepper noise, Gaussian noise, and Poisson noise to an image.

To test the effectiveness and robustness of the algorithm, we designed a testing experiment. In this experiment, we divided 260 images into three categories: low, medium, and high concentrations (low concentration: 0–50 μg/mL, medium concentration: 80–500 μg/mL, and high concentration: 800–1500 μg/mL). We randomly applied salt-and-pepper noise, Gaussian noise, and Poisson noise to the images, as shown in Figure 13. Subsequently, we tested these noisy images using the algorithm proposed in this paper. At the same time, we tested the accuracy of the algorithm on the samples without any noise added. The test results are shown in Table 3.

After adding noises, the accuracy of the algorithm in detecting light-colored test strips with a low concentration drops from 88% to 71%. However, it still maintains a high accuracy for test strips with medium and high concentrations. This indicates that the algorithm performs better in detecting test strips with darker colors and higher concentrations. Despite the added noises, it still achieves an accuracy of 85% in detecting the test strips. This demonstrates the effectiveness of the algorithm, but further improvements are needed to enhance its ability to detect targets with low concentrations.

Traditional image processing and machine learning methods, like KNN, CNN, and U-net, are widely used to analyze and detect LFIA images, but they require large training data sets and are sensitive to parameter changes, thus limiting their practical use. To address these issues, this study proposed a quantitative detection method for LFIA images based on image processing technology, which combines multiple image processing techniques and mathematical methods to effectively optimize LFIA imaging, thereby improving the accuracy and reliability of detection. The specific performance of the proposed algorithm and other common algorithms is shown in Table 4.

A comparison was made on the performance of several algorithms in various applications. The KNN classifier developed by Hyun Jung Min et al. achieved an accuracy of 95.56% in the quantification of Salmonella LFIA images with a large sample size of 1500 [19]. Huang Lei et al., using convolutional neural network (CNN), achieved an accuracy of 92% when detecting images of performance-enhancing drugs with a small sample size of 120 [20]. Qi Qin et al. adopted the U-net algorithm in deep learning and achieved the highest accuracy of 97.46% with 942 samples [21]. Guanao Zhao et al. proposed a multi-strip detection algorithm using image processing, which achieved an accuracy of 94.5% [22]. Compared to traditional image processing algorithms, the accuracy is slightly lower than KNN and U-net but slightly higher than CNN with insufficient sample size. The proposed algorithm shows better performance in cases with smaller sample sizes. Additionally, the proposed algorithm can be trained faster and demonstrates more stable performance in fitting.

## 4. Conclusions

We developed an image processing-based quantitative lateral flow immunoassay detection method. A dark box was designed to control the lighting conditions, and the accuracy of the algorithm under different lighting conditions was analyzed. Different models of smartphones were used to take photos, and the differences in the images were compared. An intelligent smartphone algorithm was designed and applied to a mobile app. The algorithm focuses on intelligently finding the optimal threshold to segment images. This method first identifies the control line with the most distinct color and then uses various factors, such as pixel mutation position, control line position, and width, and actual design distance of the test strip as the discriminant factors to intelligently locate the lighter-colored test line. The algorithm achieves an accuracy rate of 94.23% when tested on 260 test strips. After extracting the characteristic values, a mathematical model based on SVM was selected to fit the characteristic values that best correlate with concentration, yielding an *R*^2^ value of 0.99492. To test the sensitivity, robustness, and effectiveness of the algorithm, the images were divided into three categories based on sample concentration, and random noise was added for testing. It was found that the proposed algorithm maintains an accuracy rate of over 90% for detecting medium-to-high-concentration test strips with darker colors, and there is a slight decrease in performance for low-concentration test strips, but the overall accuracy rate still reaches 85%. The detection algorithm proposed in this study was compared with popular neural network algorithms, such as KNN, CNN, and U-net, and traditional image processing algorithms. It was found that the proposed algorithm can achieve comparable accuracy to the neural network algorithms with a small sample size. It also exhibits higher stability and lower algorithm complexity. 

However, there are still significant limitations to the algorithm proposed in this study. Only three models of smartphones were used in the study, and more lighting conditions need to be compared. The algorithm also needs to be adjusted when testing strips of different specifications. After adding noise to lighter-colored test strips for testing, it was found that the detection accuracy decreases significantly, and efforts need to be made to improve the accurate measurement of low-concentration test strips. This study focuses on a sample of 260 SAA immunoassay test strips, which is still insufficient, and more samples need to be added to increase persuasiveness.

Finally, this algorithm is currently suitable for personal rapid testing. However, in the future, it could be used in broader application scenarios, such as large-scale rapid testing in hospitals and community health centers, providing better technical support for disease prevention and control. In addition, the algorithm could also be applied to other types of detection, such as chemiluminescence assays [23] or enzyme-linked immunosorbent assays [24], with potential application prospects and development potential.

## Figures and Tables

**Table 1:** Camera parameters of the selected phones.

Phone Model	Camera Pixels (Million)	Aperture	Sensor Size (Inches)
Xiaomi 11	10,800	F/1.9	1/1.33
iPhone 12	12,000	F/1.6	1/1.76
Huawei P30	40,000	F/1.8	1/1.7

**Table 2:** Randomly selected five groups of sample reading values.

Concentration (μg/mL)	0	5	10	30	50	80	100	300	500	800	1000	1300	1500
1	0	0.0774	0.1297	0.0873	0.1784	0.2187	0.4009	0.9596	1.0008	1.3142	1.4978	1.9040	1.7000
2	0	0.0626	0.0824	0.1343	0.1438	0.1348	0.3498	0.9069	1.2781	1.4234	1.4498	2.0319	2.0939
3	0	0.0927	0.0663	0.0829	0.1249	0.1857	0.2821	0.7958	1.3111	1.5778	1.4626	1.5254	1.6717
4	0	0.0477	0.0367	0.1586	0.1584	0.1992	0.3389	0.8353	1.0767	1.3373	1.6317	1.4284	1.6454
5	0	0.0116	0.0314	0.0967	0.1352	0.2375	0.6874	0.8599	0.9237	0.9765	1.1935	1.2317	1.3246

**Table 3:** Test accuracy for different concentrations.

Accuracy	Low-Concentration Samples (%)	Medium-Concentration Samples (%)	High-Concentration Samples (%)	Total Accuracy (%)
No noise added	88	96.25	100	94.23
Noise added	71	90	97.5	85

**Table 4:** Performance of common algorithms.

Algorithm	Accuracy (%)	Sample Size	Goodness of Fit
This paper	94.1	205	0.995
KNN	95.56	1500	0.960
CNN	92	120	0.987
U-net	97.46	942	>0.9
Image Processing	94.5	N/A	0.993

## Data Availability

The data presented in this study are available from Xincheng Jiang upon request.
